# The Damage Capacity of *Mahanarva spectabilis* (Distant, 1909) (Hemiptera: Cercopidae) Adults on *Brachiaria ruziziensis* Pasture

**DOI:** 10.1155/2013/281295

**Published:** 2013-12-15

**Authors:** Tiago Teixeira Resende, Alexander Machado Auad, Marcy das Graças Fonseca, Fausto Souza Sobrinho, Dayane Ribeiro dos Santos, Sandra Elisa Barbosa da Silva

**Affiliations:** ^1^Embrapa Dairy Cattle Research Center, Rua Eugênio de Nascimento, 610 Dom Bosco, 36038-330 Juiz de Fora, MG, Brazil; ^2^Depatment of Entomology, Federal University of Lavras, Campus Universitário, 37200-000 Lavras, MG, Brazil

## Abstract

The aim of this study was to determine the damage caused by adult *Mahanarva spectabilis* (Distant, 1909) (Hemiptera: Cercopidae) on *Brachiaria ruziziensis* (Germain & Evard) under field conditions. A total of 0, 4, 8, 12, or 16 *M. spectabilis* adults per plot were maintained for 6 days. Thereafter, the insects were removed from the plant, and the following parameters were evaluated: chlorophyll content, damage score, dry as well as fresh weights, percentage of shoots' dry matter, and the forage's ability to regrow. The chlorophyll content was significantly reduced; the damage score and percentage of dry matter in plants increased depending on the increased insect infestation density after 6 days of exposure. In contrast, no change was observed on the *B. ruziziensis* fresh and dry weights as well as the regrowth capacity depending on the *M. spectabilis* infestation densities. Attacks by 8 adult *M. spectabilis* per clump of *B. ruziziensis* with an average of 80 tillers for 6 days were sufficient to reduce the chlorophyll content and the functional plant loss index. This density can be a reference for spittlebug integrated management in *Brachiaria*.

## 1. Introduction

In the 1970s due to the emergence of forage species with a high adaptability to climate and low soil fertility, livestock was expanded in Brazil [1]. Currently, cattle livestock is responsible for over 44% of the national cattle herd, which use grown pastures as their primary food source [2]. Extensive monoculture of such grasses favors development of high spittlebug populations [3].

Spittlebugs from the genus *Mahanarva* (Hemiptera: Cercopidae) cause serious damage in pastures and threaten meat production by compromising the forage supply [4, 5]. They have been reported in several regions and are widely distributed in south and central America [6]. The attacks by spittlebugs may even kill the grass depending on the time of the year and population density [7]. According to Thompson [8], the damage caused by spittlebugs generates losses of 840 to 2100 million dollars per year worldwide. Even though nymphs cause damage to forage, adult damage is more severe due to the toxic salivary excretions left in the shoots during feeding [9–14]. According to Byers and Wells [9], the toxic saliva injected during adult feeding interferes in the photosynthetic activity.

Valério and Nakano [11] observed a direct relationship between exposure time and damage symptom intensity in *Brachiaria decumbens* that were under attack by adult *Notozulia entreriana* (Berg 1879). Adopting a simulation model to quantify the economic impact of adult *N. entreriana*, Holmann and Peck [15] observed a drastic reduction in *B. decumbens* carrying capacity and estimated that 10 adult spittlebugs can reduce the cultivated pasture stocking rate, which significantly contributes to increased production costs.

Research has been directed towards spittlebug control through forage resistance to nymphs. However, López et al. [14] concluded that adult spittlebugs are a major threat to *Brachiaria* hybrids with high levels of antibiosis resistance to the nymphs.

According to Kain and Atkison [16], the main problem associated with studies that assess loss promoted by insects in pastures is converting quantitative and qualitative loss of grassland into livestock production and, consequently, establishing economic damage level. In this context, determining effects of adult *M. spectabilis* attacks on *B. ruziziensis* is an important tool that will assist in an integrated management of this insect pest. Thus, the aim of this study was to determine the damage caused by adult *M. spectabilis* on *B. ruziziensis* under field conditions.

## 2. Materials and Methods

### 2.1. Plants and Insects

The research was conducted at the Embrapa Dairy Cattle research experimental field, which is located in Coronel Pacheco, MG, Brazil (21°33′22′′ south and 43°16′15′′ west). During the experiment, the mean temperature was 27.8°C (maximum: 46.4°C and minimum: 20.2°C) with a 76.9% relative humidity (maximum: 98.2% and minimum: 24.3%). These parameters were recorded by a data logger every 2 minutes and transferred to software (Hoboware); these values were used to determine the average for the experimental period. Twenty days before beginning the experiment, the *B. ruziziensis* pasture was cut 15 cm above ground level to standardize plant height. Each experimental plot included a forage clump with a 30 cm mean height, 88.52 tillers, and 33.23 SPAD units (chlorophyll content). *M. spectabilis* adults were collected in a pasture distal to the experimental area. These insects were maintained in cages until they were used in the experiment.

### 2.2. Experiment

The randomized block design included 5 infestation densities and 8 repetitions. Each plot comprised a *B. ruziziensis* clump protected by a metal frame cage (70 × 40 × 40 cm) covered with organza fabric. The experimental plots were installed equidistant (5 × 2 m) and covered with 400 m^2^ meters of grassland. Inside each cage were 0, 4, 8, 12, and 16 *M. spectabilis* adults. The dead insects were replenished daily to maintain a constant *M. spectabilis* density for 6 days. Thereafter, the insects were removed and evaluated for the following parameters: chlorophyll content, damage score, dry as well as fresh weights, percentage of dry shoot matter, and the forage regrowth ability.

The chlorophyll content was measured using the apparatus Minolta SPAD 502 OL (Konica Minolta Sensing, Osaka, Japan) before infestation as well as 3 and 6 days after onset of infestation in three leaf blades from one tiller of the plant. According to Diaz-Montano et al. [17] the chlorophyll meter SPAD-502 is an important device used to measure chlorophyll loss by sucking insects. The mean chlorophyll content in the three leaf blades from each plant was calculated.

After 6 days of infestation, the damage percentage score in the shoots from each plant was assigned by three evaluators that followed the damage scale from 1 through 5 proposed by Cardona et al. [18]. Furthermore, the damage scores were classified based on scores developed by Pabón et al. [19] as follows: scores 1 and 2—the grass tolerates insect attack; scores between 2.1 and 3—intermediate tolerance; and scores over 3—susceptible to insect attack.

After 6 days, the plants with different infestation densities were cut at the soil level, and their leaves and stems were weighed for the fresh weight. These materials were dried at 55°C for 72 hours, and after this period, they were weighed to record the dry weight. The percentages were then calculated for the dry matter and the functional plant loss index (FPLI), which were proposed by Morgan et al. [20] and modified by Panda and Heinrichs [21]. This index is calculated based on the damage score (DS), dry weight of uninfested plants (DWUP), and dry weight of infested plants (DWIP), as follows: FPLI (%) = [1 − (DWIP/DWUP) × (1 − DS/5)] × 100, and this method is considered a useful tool for quantifying tolerance [22].

To evaluate the forage regrowth ability with adult spittlebugs, the metal frame of each cage was maintained for 30 days without organza fabric covering. The number of shoots in each clump was evaluated 15 and 30 days after cutting.

### 2.3. Statistical Analysis

The average chlorophyll content in the three leaf blades from each plant, damage score, fresh and dry shoot weights, dry matter percentage, and forage regrowth ability were compared using analysis of variance, and when significant (*P* ≤ 0.05), regression analyses were performed for the spittlebug infestation density. The average chlorophyll content was compared using the Tukey test to assess the effect of 3 and 6 days of forage exposure to adult spittlebugs (*P* ≤ 0.05).

The analyses were performed using the program SISVAR 5.1 [23] (Federal University of Lavras, MG, Brazil). The Pearson correlation between the chlorophyll content and damage score was generated using BioEstat 5.0 [24] (Federal University of Pará, Pará, Brazil).

## 3. Results

**Figure 1:**
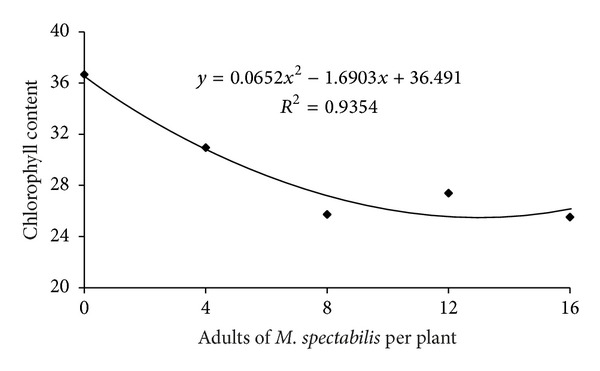
Relationship between infestation levels of adult *M. spectabilis* and chlorophyll content (SPAD unit) in *B. ruziziensis*.

In the first evaluation, which was conducted prior to forage infestation by *M. spectabilis* adults, the chlorophyll content was the same (*F* = 0.191, *P* = 0.9427) regardless of the future infestation level, which demonstrates plant standardization. This standardized plant behavior was reproduced in the second evaluation, which was performed 3 days after the plants were exposed to insects (*F* = 2.13, *P* = 0.08). In the subsequent evaluation, which was performed 6 days after infestation, the forage chlorophyll content significantly decreased due to an increase in the *M. spectabilis* infestation (*F* = 7.28, *P* < 0.0001), which indicates a quadratic regression curve for this exposure time (Figure 1).

**Figure 2:**
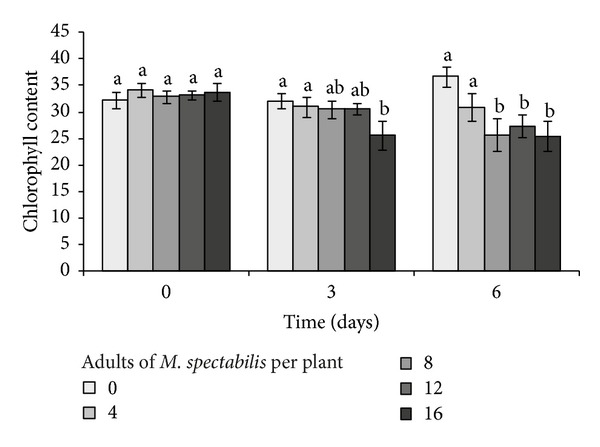
Relationship between chlorophyll content (SPAD unit) of *B. ruziziensis* and exposure time (0, 3, and 6 days) at different infestation levels of adult *M. spectabilis*. Mean values followed by the same letter within the levels of infestation did not differ by Tukey test.

After the exposure, the plant chlorophyll content did not change in plants exposed to 4 adult spittlebugs (*F* = 1.09, *P* = 0.33) and those with no contact to the insect pest (*F* = 2.25; *P* = 0.10). In contrast, there was a significant reduction in forage chlorophyll content after 6 days of exposure to 8 (*F* = 4.43, *P* < 0.01) and 12 insects (*F* = 2.89, *P* < 0.05) as well as for plants exposed for 3 days to 16 insects (*F* = 7.42, *P* < 0.001) (Figure 2).

**Figure 3:**
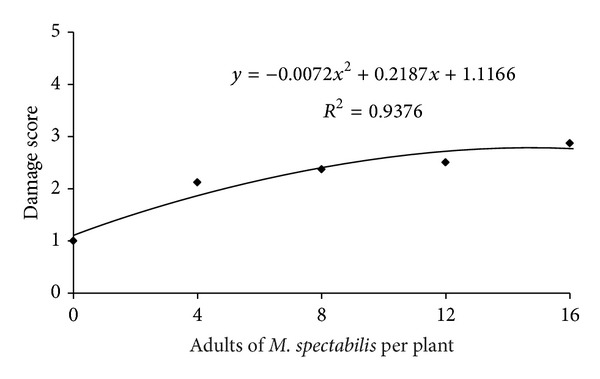
Relationship between infestation levels of *M. spectabilis* adults and damage scores for *B. ruziziensis* over 6 days.

For the damage score, higher *M. spectabilis* infestation levels yielded greater damage to plants exposed to spittlebugs for 6 days (*F* = 18.53, *P* < 0.0001) (Figure 3). Notably, under field conditions, *B. ruziziensis* had an intermediate tolerance to be attacked by 4, 8, 12, and 16 *M. spectabilis* adults for 6 days based on converting the damage score parameters into the tolerance established by Pabón et al.[19]. Further, the damage scores inversely correlate with the forage chlorophyll content upon a day exposure to *M. spectabilis* adults (*r* = −0.44, *t* = −33.48, *P* = 0.0018).

**Figure 4:**
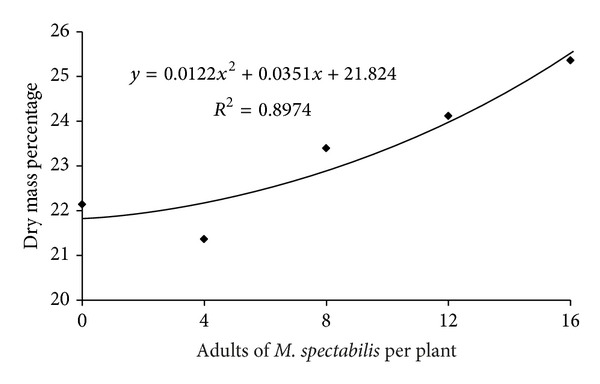
Relationship between infestation levels of *M. spectabilis* adults and dry mass percentage of the *B. ruziziensis*.

For the functional plant loss index, there was no significant difference in this ratio among infestation densities (*F* = 1.613, *P* = 0.208). However, only four and eight adult *M. spectabilis* per plant were sufficient to generate a functional plant loss index greater than 45.3% and 60%, respectively, in *B. ruziziensis*; however, for 16 adults, such a loss reached 61.9%. Likewise, increasing *M. spectabilis* densities did not significantly alter the fresh (*F* = 2.06, *P* = 0.113) and dry weights (*F* = 1.416, *P* = 0.2546) in plants exposed to the adult insects for 6 days. However, the increased infestation density caused a significant increase in the dry matter percentage of plant (*F* = 2.798, *P* = 0.0451) (Figure 4).

The tiller number in plants was equal in the assessments performed 15 (*F* = 0.565, *P* = 0.69) and 30 (*F* = 0.432, *P* = 0.78) days after cutting the forage shoots that were under different insect densities for 6 days.

## 4. Discussion

The *B. ruziziensis* chlorophyll content decreased depending on the *M. spectabilis *density and time the plant was exposed to the insect pest. Similar results were observed in Ni et al. [25] wherein attacks by *Blissus leucopterus* significantly reduced the chlorophyll content and, consequently, the photosynthetic rate in certain forage millet genotypes under field conditions; those authors reported that such a reduction decreased the leaf area and, consequently, reduced the accumulated biomass. Similarly, Wang et al. [26] observed reductions in chlorophyll content due to an attack by herbivores, which may negatively affect plants' photosynthetic capacities. This observation is concerning for forage because the forage rest period is calculated according to its capacity of regeneration in rotational grazing system, where according to Martha Júnior et al. [27], the pasture is subjected to alternating grazing and rest periods. According to the results herein, 8 and 16 *M. spectabilis* adults that attack forage clump for 6 or 3 days, respectively, are sufficient to reduce forage chlorophyll content; therefore, one can infer that a longer rest period for *B. ruziziensis* is necessary for increasing spittlebug infestation densities, given the plants' reduced photosynthetic rates, which could jeopardize the fodder supply for animals. This reduced supply should be even higher because pastures with damage symptoms from spittlebugs are less palatable, according to Valério and Nakano [11].

The plant damage scores herein were lower than reported by Cardona et al. [18] for *B. ruziziensis* (4.8); Cardona et al. [28] for *B. decumbens* (3.7) infested with 12 and 5 *Aeneolamia varia* adults per cage (6 cm in diameter by 24 cm in height); and Resende et al. [29], who observed damage scores higher than 3.5 for *B. ruziziensis* infested with different adult *M. spectabilis* densities per cage (0.40 × 0.40 × 0.80 m) in a greenhouse. Given that this study was conducted in the field, it can be inferred that this difference is related to the conditions of plant development, such as the volume of soil available to the root system for plants grown in the field, higher soil volume may yield lower damage.

Despite the observation that infestation densities of 4, 8, 12, and 16 *M. spectabilis* adults per plant caused intermediate damage in the fodder (25 to 50% leaf area was damaged), according to López et al. [14], damage by adult spittlebugs is irreversible. Thus, the portion of the forage damaged by the insect will have a compromised development capacity, which will reduce the fodder supply for animals over time and decrease milk as well as meat production. To alleviate this problem, the management tactic proposed by Painter [30] and Soares et al. [31] is viable; it uses harvest anticipation to change the timing between the presence of food and associated insects, which is a form of ecological resistance referred to as “escape from the host.” Thus, the *B. ruziziensis* pastures infested by adult *M. spectabilis* may be grazed early in the infestation, which would target the forage for animal use before the plants show damage symptoms caused by an insect attack, which may ensure forage regrowth.

The inverse relationship observed herein between the forage damage scores and chlorophyll content upon exposure to *M. spectabilis* adults was similar to the results by Resende et al. [29] who observed a high negative correlation between the damage score and chlorophyll content for *B. ruziziensis* infested with *M. spectabilis* adults in a greenhouse, and López et al. [14] who reported a high correlation between the damage score and percentage of chlorophyll loss in *Brachiaria *genotypes infested with adult spittlebugs.

The functional losses observed herein were lower than those reported by Resende et al. [29], who found that attacks by 12 *M. spectabilis* adults generated a functional plant loss index over 75% of *B. ruziziensis* in a greenhouse. Values higher than those herein were also demonstrated by López et al. [14] in *B. ruziziensis*, who recorded index of 93.4 and 100% after attacks by *A. varia* and *Z. carbonaria* adults, respectively. The functional plant loss index measures the plants' tolerance to insects [20, 21], and according to López et al. [14], it is the best index for estimating *Brachiaria* tolerance to spittlebugs.

The differences from attacks by adult spittlebugs in the aforementioned trials are attributed to the different conditions of plant development. The study herein is the only study to assess the impact of adult *M. spectabilis* under field conditions with no physical limitations on land area explored by roots, which ensures greater energy reserves in this region of plants. Thus, plants tend to better tolerate insect pest attacks under field conditions than in the greenhouse.

The increased *B. ruziziensis* dry matter percentage due to the enhanced *M. spectabilis* infestation density herein was also demonstrated by Resende et al. [29] in *B. ruziziensis* 10 days after exposure to 24 *M. spectabilis *adults in a greenhouse. Those authors attributed the increased dry matter percentage to the reduced fresh weight from insect damage. The increased dry matter percentage was also reported for *B. decumbens* infested with *Zulia entreriana* adults [11] in alfalfa; red clover attacked by *Philaenus spumarius* [32, 33]; and *Digitaria decumbens* infested with *Prosapia bicincta* [34].

Despite the shoot damage from the *M. spectabilis* attack, such damage levels did not affect the *B. ruziziensis* regrowth ability under field conditions. In contrast, Resende et al. [29] observed a significantly reduced number of *B. ruziziensis* shoots infested with 12 or more *M. spectabilis *adults in a greenhouse. This observation is attributed to differences in the fodder development conditions considering that herein the plants were grown directly in the ground without physical constraints to root system development. Because differences were not observed in the fresh weight and forage regrowth after the pest insect attack, tactics for using such forage should be adopted early during infestation before the more advanced symptoms of attack by adults. Among these tactics, the forage by grazing animals or even cutting and storage of this material in silos to be provided to cattle during the dry season are highlighted. According to Valério [35], the forage root system may be reduced due to frequent spittlebug attacks, which results in reduced forage persistence. The plants used herein were grown under favorable conditions for extensive root development and were not previously attacked by spittlebugs. These factors were important to maintain the number of shoots after infestation; these data emphasize that proper pasture and soil fertility management guarantee forage re-establishment even after insect attacks.

## 5. Conclusion

Thus, attacks by 8 adult *M. spectabilis* per clump of *B. ruziziensis* with an average of 80 tillers for six days were sufficient to reduce the chlorophyll content and cause functional plant loss index of 60%, but it did not affect the fresh weight nor regrowth capability, which indicates that tactics for using this forage are recommended at the beginning of insect infestation. This population density can be a reference for spittlebug integrated management in *Brachiaria*.
